# Combining Gut Microbiota Modulation and Enzymatic-Triggered Colonic Delivery by Prebiotic Nanoparticles Improves Mouse Colitis Therapy

**DOI:** 10.34133/bmr.0062

**Published:** 2024-08-13

**Authors:** Hui Li, Yu Cheng, Luwen Cui, Zizhen Yang, Jingyi Wang, Zixuan Zhang, Kaiwei Chen, Cheng Zhao, Ningning He, Shangyong Li

**Affiliations:** ^1^ School of Basic Medicine, Qingdao Medical College, Qingdao University, Qingdao, China.; ^2^ Department of Abdominal Ultrasound, The Affiliated Hospital of Qingdao University, Qingdao, China.

## Abstract

The efficacy of ulcerative colitis (UC) therapy is closely connected to the composition of gut microbiota in the gastrointestinal tract. Prebiotic-based nanoparticles (NPs) provide a more precise approach to alleviate UC via modulating gut microbiota dysbiosis. The present study develops an efficient prebiotic-based colon-targeted drug delivery system (PCDDS) by using prebiotic pectin (Pcn) and chitosan (Csn) polysaccharides as a prebiotic shell, with the anti-inflammatory drug sulfasalazine (SAS) loaded into a poly(lactic-co-glycolic acid) (PLGA) core to construct SAS@PLGA-Csn-Pcn NPs. Then, we examine its characterization, cellular uptake, and in vivo therapeutic efficacy. The results of our study indicate that the Pcn/Csn shell confers efficient pH-sensitivity properties. The gut microbiota-secreted pectinase serves as the trigger agent for Pcn/Csn shell degradation, and the resulting Pcn oligosaccharides possess a substantial prebiotic property. Meanwhile, the formed PCDDSs exhibit robust biodistribution and accumulation in the colon tissue, rapid cellular uptake, efficient in vivo therapeutic efficacy, and modulation of gut microbiota dysbiosis in a mouse colitis model. Collectively, our synthetic PCDDSs demonstrate a promising and synergistic strategy for UC therapy.

## Introduction

Ulcerative colitis (UC) is a chronic inflammatory disease of the colon that is increasing in incidence and prevalence [1]. The etiology and pathogenesis of UC remain complex and unclear. However, it is closely associated with immune response imbalance and alteration of colonic barrier function caused by gut microbiota dysbiosis [2,3]. The efficacy of therapeutic drugs for UC, such as 5-aminosalicylic acid (5-ASA), immunomodulators, and biological agents, is primarily impeded by inevitable side effects. The diminished effectiveness of these drugs can be attributed to gastrointestinal tract (GIT) issues that arise following oral administration, infection risks [4] or the inconvenience associated with injectable treatments [5,6].

Oral drug delivery systems (ODDSs) offer convenient operation and minimal side effects, rendering them highly desirable for UC therapy [7]. ODDSs exhibit pH sensitivity, gastric resistance, and susceptibility to degradation by gut microbiota in the colon. These characteristics enable ODDSs to achieve elevated localized concentrations at the site of lesion, while ameliorating side effects associated with drug release in the GIT or unwarranted systemic absorption. Current studies have enabled nanoparticles (NPs) to have colonic release and target colonic epithelial/immune cells by preparing NPs by coupling hyaluronic acid (HA), calcium pectinate (CP), or lysine–proline–valine (KPV) [8–10]. Studies of the gut microbiome and host–microbiome interactions have provided important insights into the pathogenesis of inflammatory bowel disease (IBD) and clues to the development of microbiome-based diagnostics and interventions for IBD [11]. Moreover, the combination of gut microbiota modulation and medication in prebiotic-based colon-targeted drug delivery systems (PCDDSs) possesses great potential to enhance various physicochemical properties of therapeutic drugs, thereby offering a promising treatment strategy for UC [12].

Pectin (Pcn) is a dietary prebiotic widely derived from the skin and flesh of citrus fruits, apples, and the cellular wall of algae [13]. Previous studies have also demonstrated its potential in exhibiting anti-diabetic, anti-hyperlipidemic, and anti-cancer properties [13]. Our previous study demonstrated that Pcn oligosaccharides, the enzymatic product of Pcn, exert a significant impact on the prevention and treatment of acute colitis through their modulation of the gut microbiota and Treg/Th17 cell balance in mice [14]. Meanwhile, Pcn can serve as a carrier in PCDDS formulations to improve encapsulation efficiency via cross-linking, ensuring high stability in the stomach, demonstrating colon-specific swelling behavior, and undergoing absolute degradation by pectinase produced by gut microbiota [15]. The gene expression of pectinases is lower in UC patients, which partly serve as a biomarker of beneficial bacteria. Therefore, the incorporation of Pcn-based PCDDSs presents an innovative opportunity to enhance the efficacy of UC treatment through precise drug delivery and modulation of gut microbiota.

**Fig. 1.**
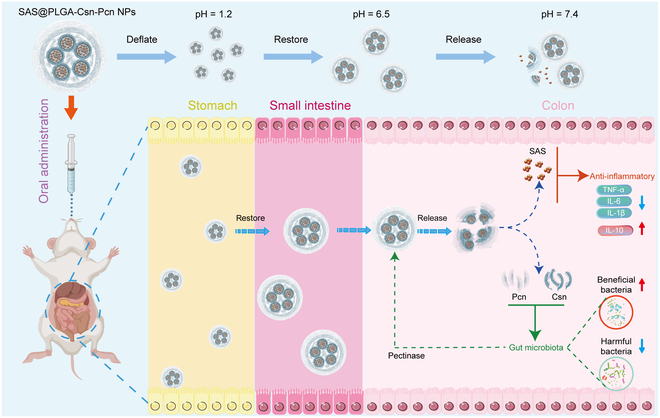
Schematic illustration of the SAS@PLGA-Csn-Pcn NPs. The Pcn/Csn shell offers pH-dependent release protection in the upper GIT. The pectinase from gut microbiota triggers PCDDS degradation and further regulates the gut microbiota ecosystem.

In this study, we developed an efficient PCDDS by using Pcn and chitosan (Csn) as a protective shell, loading sulfasalazine (SAS) into a poly(lactic-co-glycolic acid) (PLGA) core to construct SAS@PLGA-Csn-Pcn NPs as illustrated in Fig. 1. The formulated NPs exhibited significant pH-dependent characteristics for protecting anti-inflammatory drugs in the upper GIT. Meanwhile, this PCDDS demonstrated a specific colonic delivery property triggered by pectinase secreted from the gut microbiota, and the enzymatic product exhibited remarkable prebiotic properties.

## Materials and Methods

### Materials

PLGA with a lactic acid-to-hydroxyacetic acid ratio of 50:50 was procured from Aladdin (Shanghai, China). Csn and rhodamine B isothiocyanate (RBITC) were obtained from Sigma-Aldrich (St. Louis, MO, USA). Pcn with galactose uronic acid content ≥74% and SAS were purchased from Macklin (Shanghai, China). Fecal DNA extraction kit was purchased from Tiangen Biotech (Beijing, China). Antibodies for FITC-CD3 (#100204), PE-CD4 (#100407), PE/Cyanine 5-CD25 (#102010), APC-IL17A (#506915), and Alexa Fluor@647-Foxp3 (#126407) were acquired from BioLegend (San Diego, CA, USA).

### Preparation and characterization of NPs

The SAS@PLGA oil-in-water (O/W) emulsion was prepared by combining 20 mg of PLGA in 900 μl of dichloromethane with 20 mg of SAS in 100 μl of dimethyl sulfoxide, followed by addition to a 4-ml solution containing 1% poly(vinyl alcohol) and sonication for a duration of 2 min. The layer-by-layer (LBL) method was employed to encapsulate SAS@PLGA-Csn-Pcn NPs. Briefly, Csn solution (1% w/v acetic) was slowly added to the SAS@PLGA core under continuous stirring for a duration of 2 h. The mixture was then centrifuged and washed with ddH_2_O. Subsequently, the Pcn solution was gently added under continuous stirring, followed by washing and freeze-drying. The synthesis of RBITC-labeled Csn was constructed based on our previous study [16].

The formed SAS@PLGA core and SAS@PLGA-Csn-Pcn NPs were characterized by transmission electron microscope (TEM, JEM-1200 EX, Tokyo, Japan) at an acceleration voltage of 100 kV. The Zetasizer Nano ZS instrument (Malvern Instrument, Malvern, UK) was utilized for particle size and zeta potential determination via dynamic light scattering (DLS) and electrophoretic light scattering (ELS), respectively. The Alpha Fourier transform infrared (FT-IR) spectroscopy system (Bruker, Billerica, MA, USA) was employed for the KBr pressure disk technique within the frequency range of 4,000 to 400 cm^−1^.

### Encapsulation efficiency and drug loading capacity

The SAS content in the formed NPs was indirectly determined by measuring the concentration of free drug in the supernatant after 3 washes. The absorbance of the solution was measured at 359 nm by ultraviolet spectrophotometry and the free drug content was calculated according to the standard curve. The formulas for encapsulation efficiency (EE) and drug loading capacity (LC) are as follows:

$$\text{EE}\%=\frac{\text{Total}\;\text{SAS}\;\text{weight}–\text{Free}\;\text{SAS}\;\text{weight}\;}{\text{Total}\;\mathrm{SAS}\;\text{weight}}\times 100\;\left(\%\right)$$ (1)

$$\text{LC}\%=\frac{\text{Total}\;\text{SAS}\;\text{weight}-\text{Free}\;\text{SAS}\;\text{weight}}{\text{Total}\;\text{NPs}\;\text{weight}}\times 100\;\left(\%\right)$$ (2)

### In vitro drug release

To simulate the release rate of the NPs in the GIT, 3 different pH levels were employed in vitro to mimic the acidic environment of the stomach (pH 1.2), small intestine (pH 6.8), and colon (pH 7.4) following the methods described in our previous study [16]. To determine the enzyme-responsive release property of NPs, a solution containing fecal microbiota suspension at a concentration of 50 mg/ml or pectinase at a concentration of 125 mg/ml was used. The formulas for cumulative drug release are as follows:

$$\begin{array}{c} \text{The cumulative drug release}\%=\\ \frac{\text{Cumulative drug release}\;}{\text{The total amont of particle loading}\;\left(\text{mg}\right)}\times 100\;\left(\%\right) \end{array}$$ (3)

### Cell viability and cellular uptake of NPs in RAW264.7 and Caco-2 cells

RAW264.7 and Caco-2 cells were cultured in a humidified atmosphere with 5% CO_2_. The cytotoxicity of the NPs was evaluated using Cell Counting Kit-8 (CCK-8) assays. Fluorescence microscopy imaging with RBITC-labeled NPs was employed to observe and analyze the cellular uptake of NPs after a specified cultivation time. 4′,6-diamidino-2-phenylindole (DAPI) staining was performed to detect fluorescence signals.

### Animals and DSS-induced colitis model

C57BL/6J mice (6 weeks old, male, 18 to 22 g) were procured from Pengyue (Jinan, China). Three mice were randomly allocated to each cage and housed in the animal facility under standard conditions (temperature: 22 ± 2°C, humidity: 50% ± 15%, 12-h/12-h light–dark cycle). After 1 week of co-housing adaptation, the animals were randomly divided into 4 groups based on their diet (*n* = 6) and received different treatments as follows: (a) NC group: no treatment; (b) DSS group: 2.5% DSS for 1 week; (c) NPs group: NPs (20 mg/kg/day) and 2.5% DSS for 1 week; (d) 5-ASA group: 5-ASA (20 mg/kg/day) and 2.5% DSS for 1 week. In this experiment, mice were given the NPs or 5-ASA while drinking free water containing 2.5% DSS for a week to induce enteritis and observe the effect of the treatment. The single blind method was used to measure the treatment effect in different groups. The body weight was recorded daily, and the dosage of NPs and 5-ASA was adjusted based on changes in body weight. The disease activity index (DAI) scores were calculated by taking into account weight change, fecal consistency, and degree of bloody stool to assess the severity of intestinal inflammation [16] and scored according to Table S1. To analyze gut microbiota, the fecal samples were collected and stored at −80°C before the end of the experiment. After the mice were anesthetized, blood samples were collected. Colon samples were collected from dissected mice for measurement of colon length and histological analysis.

During the modeling stage, the body weight was assessed daily. All experimental procedures adhered to the guidelines outlined in the *Guide for the Care and Use of Laboratory Animals: Eighth Edition*, ISBN-10: 0-309-15396-4. The animal procedures conducted in this study have been approved by the Ethics Committee of the Medical College of Qingdao University (QDU-AEC-2023387). Surgeries on animal subjects were performed under anesthesia to minimize suffering.

### Adhesion experiment

The RBITC-labeled NPs (20 mg/kg/day in 100 μl) were administered orally to monitor the distribution of NPs in colon tissues at various time points (0, 6, and 12 h) for mice in the NC group and DSS group. After the mice were euthanized, frozen slices of colitis tissues were prepared, stained with DAPI, and observed using a fluorescence microscope.

### Histological evaluation

The histopathological changes in colon tissues were analyzed by hematoxylin and eosin (H&E) staining and the expression of mucin was assessed using alcian blue straining. For detailed methods, refer to our previous study [17].

### Flow cytometry analysis

Cell suspensions were collected from spleen and cells were re-suspended in PBS after lysis of the red blood cells. Prior to Th17 staining, the cells were counted and then stimulated with PMA mixture for 6 h. Treg cells and Th17 cells were stained according to our previous study [18]. After staining, the cells were detected using flow cytometry (BD FACSVerseTM, NJ, USA)

### Measurement of inflammatory cytokines in serum

Blood samples were centrifuged at 3,000 rpm for 15 min to obtain the serum samples. Inflammatory cytokines were measured using ELISA kits (Abclonal, Wuhan, China) according to instructions.

### Gut microbiota analysis

The V3–V4 region of the bacterial 16S rRNA was amplified using polymerase chain reaction and the sequencing library was generated. The amplified DNA was then sequenced using the NovaSeq 6000 platform (Shenzhen, Guangdong, China). The NovoMagic cloud platform (Shenzhen, Guangdong, China) was then used for further analysis.

### Human participants

Previously published metagenomic data from the IBD cohort generated as part of the HMP2 project (https://ibdmdb.org/) was used to explore differences in microbiota profiles between UC patients and healthy individuals. The SPSS software was utilized for comprehensive statistical analysis, and charts and graphs were generated. Proper authorization has been obtained for the use of this dataset and measurements have been taken to comply with ethical and copyright regulations related to its use.

### Statistical analysis

GraphPad Prism 9.3 was used for image construction and statistical analysis. Student’s *t* tests were used to evaluate significant differences between 2 groups, and one-way ANOVA was used to evaluate differences between multiple groups. *P* < 0.05 is considered statistically significant.

## Results

### Preparation and characterization of SAS@PLGA-Csn-Pcn NPs

**Fig. 2.**
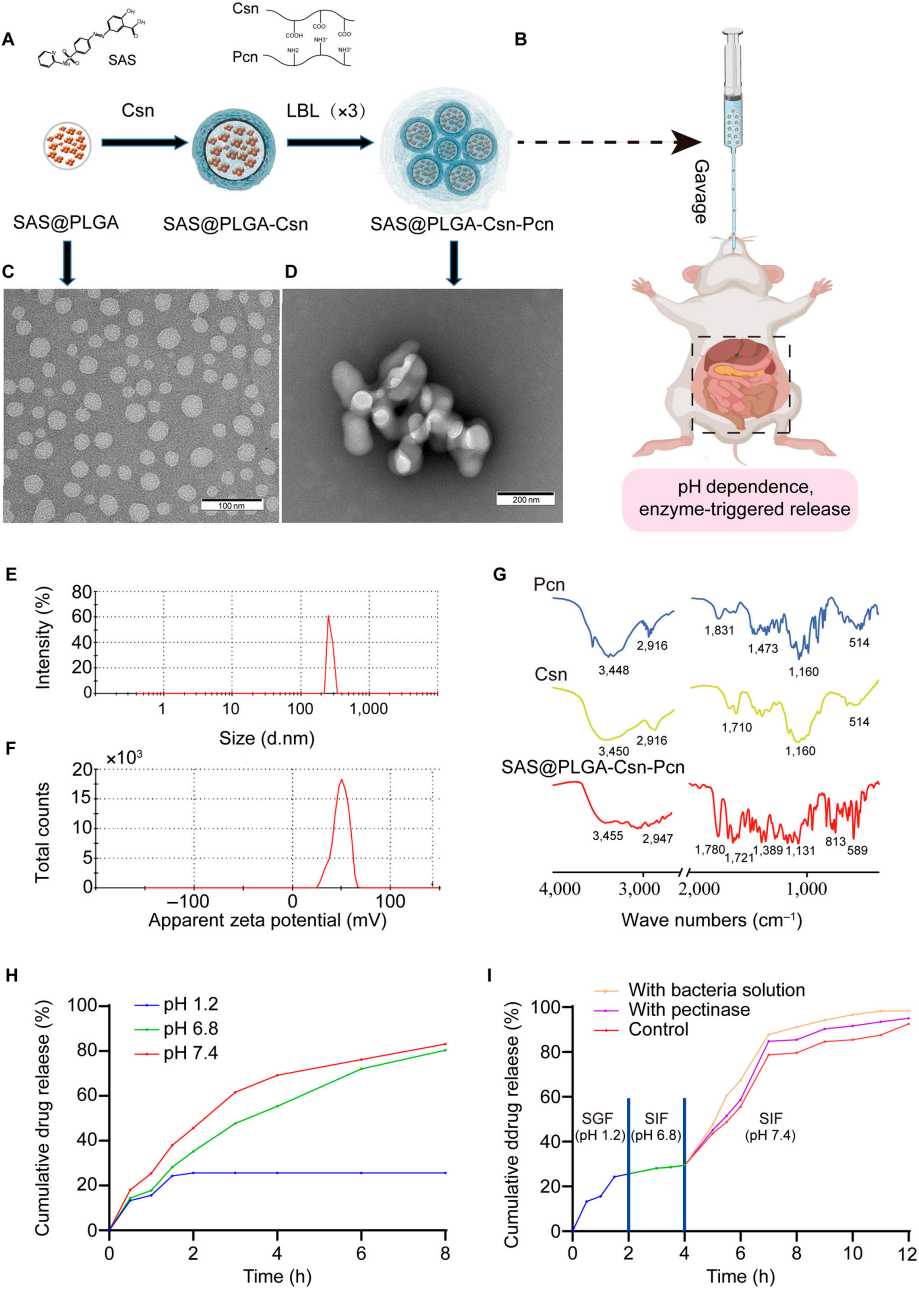
The preparation and characterization of SAS@PLGA-Csn-Pcn NPs. Preparation scheme of NPs (A) and oral scheme for colitis mouse (B). TEM images of SAS@PLGA core (C) and the formed NPs (D). Size distribution profiles (E) and zeta potentials (F) of NPs. (G) FT-IR of Csn, Pcn, and SAS@PLGA-Csn-Pcn NPs. (H) Release curves of NPs at different pH conditions. (I) Release curves of NPs with bacterial solution or pectinase in simulated digestive system. Experiments were performed in triplicate and repeated 3 times.

The preparation scheme of SAS@PLGA-Csn-Pcn NPs and oral gavage for colitis mouse is shown in Fig. 2A and B, respectively. The morphological characteristics of the SAS@PLGA core and the formed NPs were analyzed by TEM**.** The SAS@PLGA core exhibited a tendency to undergo self-aggregation in the O/W solution, resulting in the formation of simple micelles with a particle size ranging from 20 to 50 nm (Fig. 2C). Following 3 cycles of the LBL technique, SAS@PLGA was successfully encapsulated within Pcn shells (Fig. 2D). The resulting NPs had an average particle size of 269.41 ± 7.55 nm (Fig. 2E). The zeta potential of NPs exhibited a positive charge of 49.7 ± 1.35 mV (Fig. 2F), which facilitates adhesion to inflammatory sites. As shown in Fig. 2G, the FT-IR spectra of Pcn exhibited a broad band at 3,448 cm^−1^ attributed to O-H stretching, while the band at 2,916 cm^−1^ was due to C-H stretching [19]. The polymer displayed a characteristic band at 1,831 cm^−1^ owing to the presence of an ester carbonyl C=O group, and bands at 1,473 cm^−1^ correspond to COO^**-**^ group stretching. Additionally, the absorption band was observed at 1,160 cm^−1^ due to the presence of ether linkage and glycosidic bond in Pcn. Finally, an absorption band located at 1,060 cm^−1^ could be attributed to C-H bending. The FT-IR spectrum absorption peaks of the formed NPs suggested that the amine group of Csn underwent a reaction with the carboxyl group of Pcn.

The EE and LE of generated NPs were 49.7% and 15.78%, respectively. As shown in Fig. 2H, SAS has the highest release rate at pH 7.4, and only 25.66% of SAS was released at pH 1.2 within 12 h. The strong electrostatic interaction between the carboxyl group of Pcn and the amino group of Csn causes NPs to shrink at low pH, providing protection to SAS in the GIT and the stomach environment. Meanwhile, the release of SAS from NPs mixed with gut microbiota solution (50 mg/ml) or pectinase (125 mg/ml) exhibited a significant increase within 8 h, reaching about 90% (Fig. 2I). Specifically, 5 mg of NPs contained 0.789 mg of sulfasalazine. Within 8 h, 0.628 mg of sulfasalazine was released from the blank group, 0.719 mg from the extract group, and 0.674 mg from the pectinase group. These results indicated that NPs are pH sensitive and responsive to gut microbiota-produced pectinase for controlled release.

### In vitro cellular uptake and cytotoxicity of SAS@PLGA-Csn-Pcn NPs

**Fig. 3.**
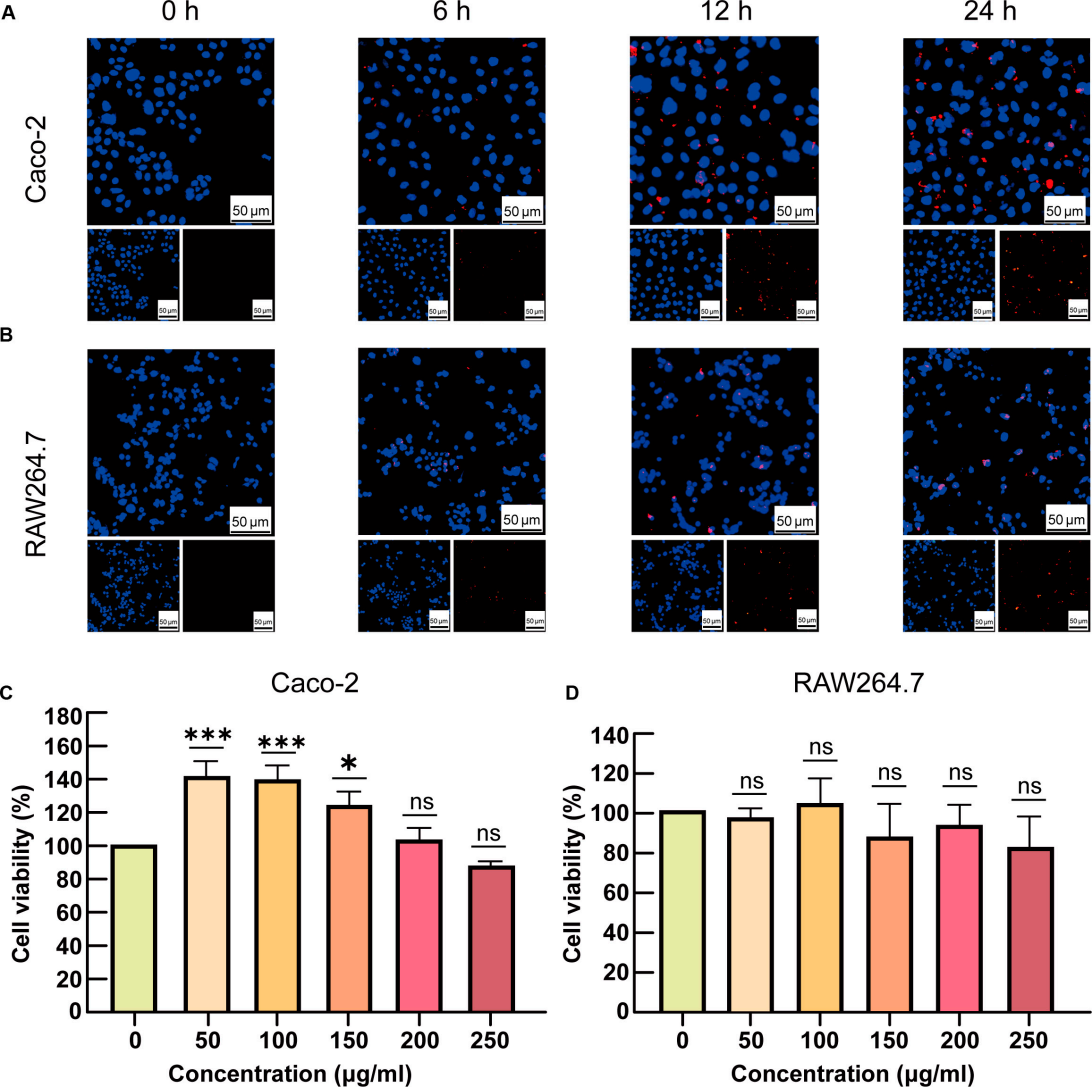
Cellular uptake and cytotoxicity of SAS@PLGA-Csn-Pcn NPs in vitro. Cellular uptake of NPs by Caco-2 cells (A) and RAW264.7 cells (B). Scale bars represent 50 μm. Cytotoxicity evaluation of NPs at various concentrations on Caco-2 (C) and RAW264 cells (D). Cells were exposed to RBITC-labeled NPs (red), and the fixed nuclei were observed with DAPI staining (blue). Compared with the indicated group. *P < 0.05, ***P < 0.001; ns, no significance. Experiments were performed in triplicate and repeated 3 times.

To determine the in vitro cellular uptake of synthetic PCDDSs, SAS@PLGA-Csn-Pcn NPs were synthesized utilizing RBITC-labeled Csn and co-incubated with macrophage cells (RAW 264.7 cells) and intestinal epithelial cells (Caco-2 cells) to enable co-localization analysis of red fluorescence (RBITC) with blue fluorescence (DAPI). As shown in Fig. 3A and B, the cellular uptake efficiency in both Caco-2 cells and RAW264.7 cells gradually improved over time. In RAW264.7 cells, the RBITC-labeled NPs efficiently enter into RAW264.7 cells and accumulate in significant quantities near the nucleus. However, during the adsorption process of Caco-2 cells, we observed a significant accumulation of RBITC-labeled NPs at a specific distance from the nucleus, posing challenges for their removal from the cell culture medium. This observation strongly suggests predominant localization on the surface of Caco-2 cells.

Biocompatibility is a crucial aspect to consider when evaluating the potential of using NPs for UC treatment. To evaluate potential cytotoxic effects, the influence of NPs on RAW264.7 and Caco-2 cells was quantified using the CCK-8 assay (Fig. 3C and D). The results showed that NPs did not exhibit significant cytotoxic effects at all concentrations for RAW264.7 cells. Interestingly, NPs exhibited a significant proliferative effect on Caco-2 cells at medium to low concentrations, potentially attributed to the capacity of NPs for cell surface adhesion. These findings indicate that NPs demonstrate outstanding in vitro biocompatibility, thereby supporting their safe use as drug delivery carriers.

### In vivo tissue uptake and Treg/Th17 cell populations

**Fig. 4.**
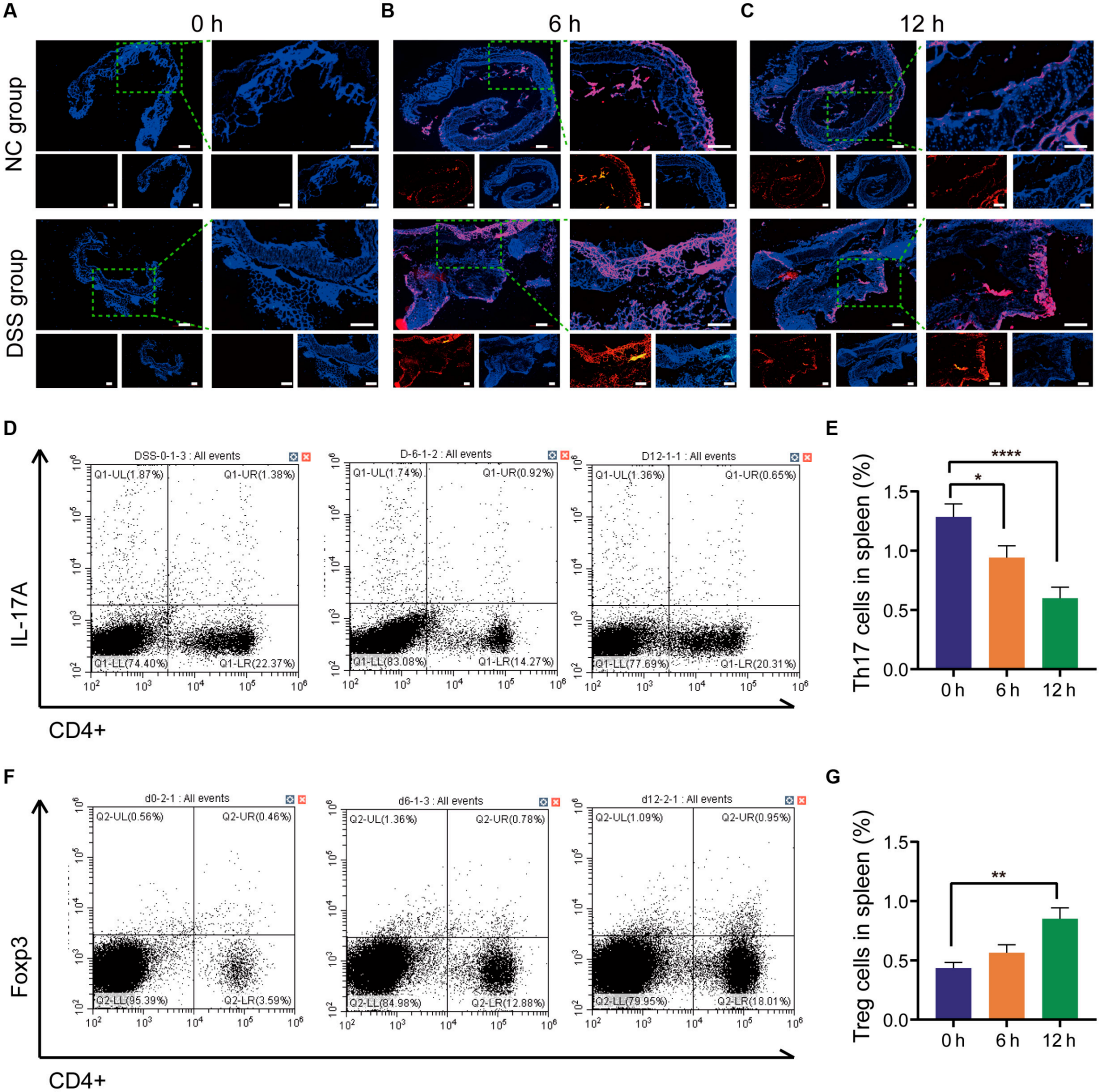
Efficiency of SAS@PLGA-Csn-Pcn NPs uptake and effect on Treg/Th17 cell balance in colitis tissues. (A to C) Fluorescence microscopy profile of healthy colonic tissue or colitis tissue. Scale bars represent 50 μm. Representative flow cytometry images of Th17 (CD3^+^CD4^+^IL17A^+^) (D) and Treg (CD3^+^CD4^+^CD25^+^Foxp3^+^) cells (F) after oral NPs administration at 0 h, 6 h, and 12 h. Percentage of Th17 cells (E) and Treg cells (G). The NC group and DSS group (*n* = 6) were treated with oral RBITC-labeled NP administration at 0 h, 6 h, and 12 h. Blue, DAPI for nuclear staining; red, RBITC-labeled NPs. Compared with the indicated group. *P < 0.05, **P < 0.01, ****P < 0.0001.

Colitis has the potential to disrupt the gut barrier, and the increased permeability of inflammatory tissue allows nanoscale drug delivery systems to have a longer residence time in the colon, leading to selective accumulation of NPs in the inflamed tissue [20]. The specific adherence and accumulation of NPs in healthy and colitis tissues were evaluated after treatment with RBITC-labeled NPs for 0, 6, and 12 h (Fig. 4A to C). It was observed that compared to colitis colon tissue, healthy colon tissue retained relatively little NPs at 6 and 12 h, suggesting the difficulty of NPs to penetrate the overall structure of healthy colonic tissue (Fig. 4B and C). Moreover, a large number of RBITC-labeled NPs were found to accumulate and be internalized by epithelial cells, penetrating deep into the mucosa. Therefore, it is demonstrated that NPs serve as a successful delivery system capable of penetrating colitis tissues and facilitating the internalization of SAS into target cells to alleviate DSS-induced colitis.

To determine the essential effect of NPs on Treg/Th17 cell balance, spleens were collected from mice with oral NPs for flow cytometry analysis (Fig. 4D to G). The results showed that NPs increased the number of Treg cells and decreased the number of Th17 cells in the spleens of DSS-induced colitis mice. Our results indicate that NPs play a role in rebalancing the Treg/Th17 cell populations and promoting immune homeostasis. In summary, NPs have the ability to restore host immune homeostasis in DSS-induced colitis model by rebalancing the Treg/Th17 cell populations.

### SAS@PLGA-Csn-Pcn NPs alleviated DSS-induced colitis in mice

**Fig. 5.**
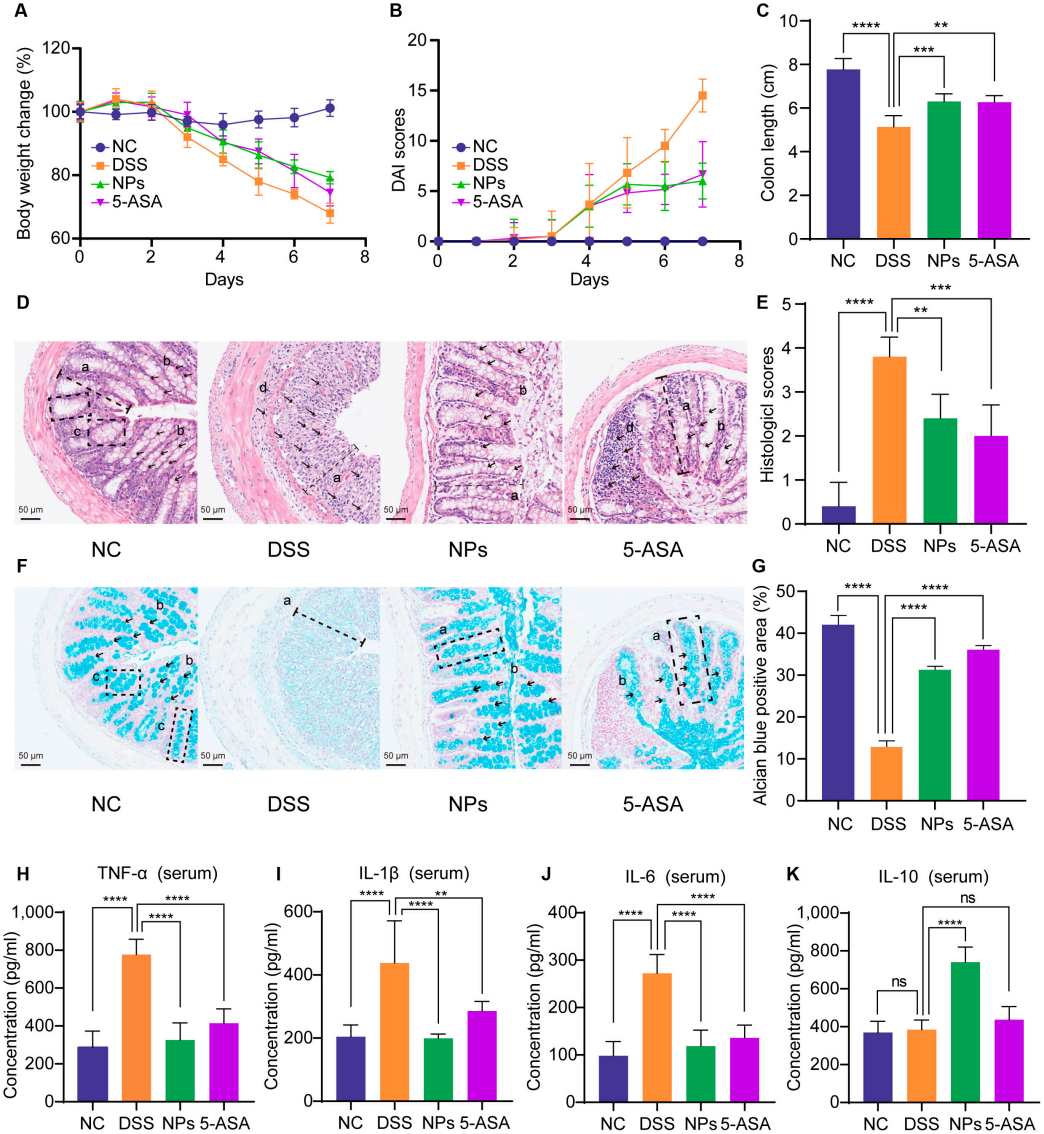
SAS@PLGA-Csn-Pcn NPs alleviated pathological lesions of DSS-induced colitis. The body weight (A) and DAI scores (B) change during experiment. (C) The statistical analysis for colon length. (D) Typical graphics of the colon sections stained with H&E. a, mucosal epithelial; b, cupped cell; c, glandular; d, inflammatory cell. (E) Histological scores of colons tissues. (F) Typical graphics of the colon sections stained with alcian blue. Plotting scale = 100 μm. (G) The positive area analysis by ImageJ. The concentrations of TNF-α (H), IL-1β (I), IL-6 (J), and IL-10 (K) in serum. *n* = 6. Compare with the indicated group, **P < 0.01, ***P < 0.001, ****P < 0.0001; ns, no significance.

To investigate the ameliorative and therapeutic effects of NPs on colitis, the DSS-induced colitis model was constructed, and 5-ASA was used as a treatment control. Compared with the NC group, DSS treatment significantly reduced the body weight of mice, while NP treatment reversed the trend of body weight loss (Fig. 5A). Colitis severity was evaluated with DAI scores [21]. As shown in Fig. 5B, DAI scores were significantly increased in DSS-treated mice during DSS induction, while NP-treated mice remarkably reduced this increase, which was consistent with changes in body weight. The LE of formed NPs was determined to be 15.78%, indicating that the potential maximum dosage of the drug (5-ASA) delivered to the colon would be 1.21 mg/kg/day. In fact, the usage of 5-ASA poses a challenge in the clinical use for UC due to the absorption issues in the small intestine [22]. Although the drug concentration of 5-ASA alone was 20 mg/kg/day, the effect of the formed NPs in alleviating colitis is similar to that of 5-ASA from the changes in body weight and DAI scores. A key indicator of UC severity is colon length; thus, changes in colon length have been calculated for 4 groups (Fig. 5C). It can be observed that both NPs and 5-ASA treatment prevented DSS-induced colon length shortening. These results indicated that NPs and 5-ASA have similar effects in alleviating DSS-induced colitis and improving the quality of life in mice.

Histological assessment of colon tissues stained with H&E or alcian blue was conducted to evaluate the protective effect of NPs on colon tissues (Fig. 5D to G). Histopathological evaluation revealed that compared with the NC group, the DSS group had greater infiltration of inflammatory cells, glandular destruction, cupped cell reduction, and mucosal epithelial necrosis, while NPs and 5-ASA could restore the pathological injury of the colon. These results suggested that NPs can improve the histological damage of the colon in DSS-induced mice.

The balance between pro-inflammatory and anti-inflammatory cytokines is critical for regulating the intestinal inflammatory microenvironment, thereby regulating the progression of UC to mucosal healing or chronic inflammation [23]. To verify the anti-inflammatory effect of NPs on colitis, we measured the concentrations of anti-inflammatory and pro-inflammatory cytokines in serum (Fig. 5H to K). Compared with the NC group, the concentrations of pro-inflammatory factors (tumor necrosis factor [TNF]-α, interleukin [IL]-1β, and IL-6) in the DSS group were significantly increased. After treatment with NPs, the concentrations of these 3 pro-inflammatory factors were significantly reduced (*P* < 0.001). The concentration of the anti-inflammatory cytokine (IL-10) in the NPs group was significantly increased (*P* < 0.05). The above results indicated that NP supplementation can significantly reduce the expression of pro-inflammatory factors and increase the expression of anti-inflammatory factors in DSS-induced colitis mice. In conclusion, NPs can not only reduce inflammation in colon tissue but also reduce the occurrence of systemic inflammation.

### SAS@PLGA-Csn-Pcn NPs regulated gut microbiota in DSS-induced colitis mice

**Fig. 6.**
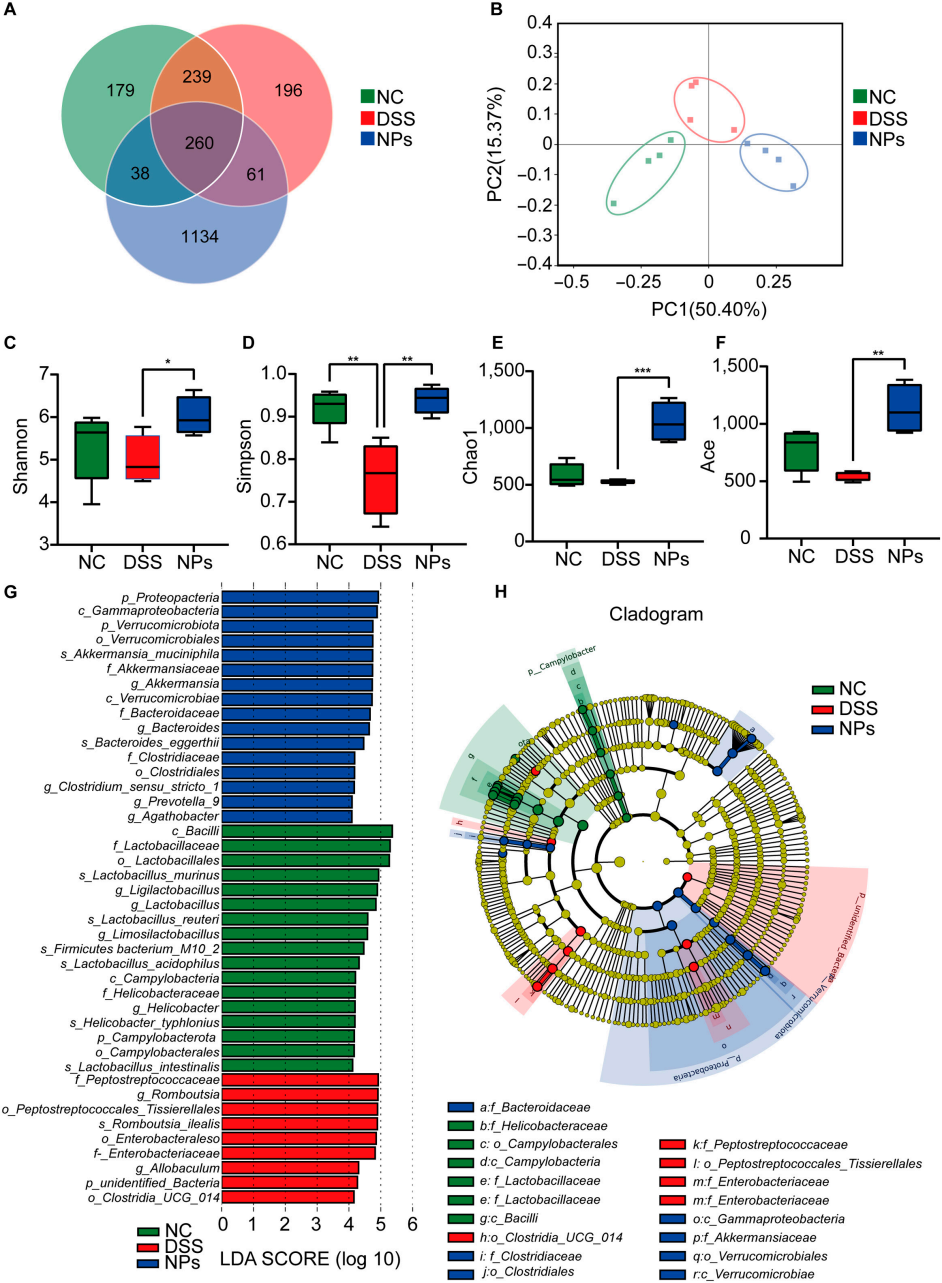
SAS@PLGA-Csn-Pcn NPs modified the composition of the gut microbiota. The fecal samples were obtained for 16S rRNA sequencing (*n* = 4). The OUT Venn diagram (A) and the diagram of principal coordinate analysis (PCoA) (B). (C to F) The change of alpha-diversity indicators. (G) Distribution histogram of LEfSe, displaying LDA score (log10) > 2 and *P* < 0.05. (H) A cladogram displaying the findings from the LEfSe analysis. Compare with the indicated group, *P < 0.05, **P < 0.01, ***P < 0.001.

The 16S rRNA sequencing study was carried out to examine whether NPs regulate gut microbiota to alleviate colitis. As shown in Fig. 6A, it can be preliminarily observed that NP treatment significantly changes the composition of gut microbiota. The weighted UniFrac principal coordinates analysis (PCoA) effectively categorized the gut microbiota composition in 3 distinct groups (Fig. 6B). Alpha-diversity was assessed by the Shannon index, Simpson index, Chao1 index, and Ace (Fig. 6C to F). The alpha-diversity index (Simpson) in the DSS group was significantly lower than that in the NC group, but there was a significant increase after NP treatment (Shannon index, Simpson index, Chao1 index, and Ace). By utilizing linear discriminant analysis (LDA) effect size (LEfSe), we determined the taxa, which could be significantly affected in the 3 groups at the family (f), genera (g), class (c), phylum (p), order (o), and species (s) levels (Fig. 6G and H). The 43 bacterial taxa with significant differences among the 3 groups were identified. Therefore, we proposed that high-abundance bacterial taxa may have potential as markers of the gut microbiome to DSS-induced colitis. Conversely, NPs revealed an increase in the abundance of *Akkermansia_muciniphila* and *Bacteroidaceae* at the family level, *Verrucomicrobiales* and *Clostridiaceae* at the order level, and *Gammaproteoacteria* and *Verrucomicrobiae* at the class level. The LEfSe study revealed significant bacterial variations in the fecal microbiota between the 3 groups. Our results indicated that the role of NPs in alleviating colitis is closely related to the regulation of gut microbiota.

**Fig. 7.**
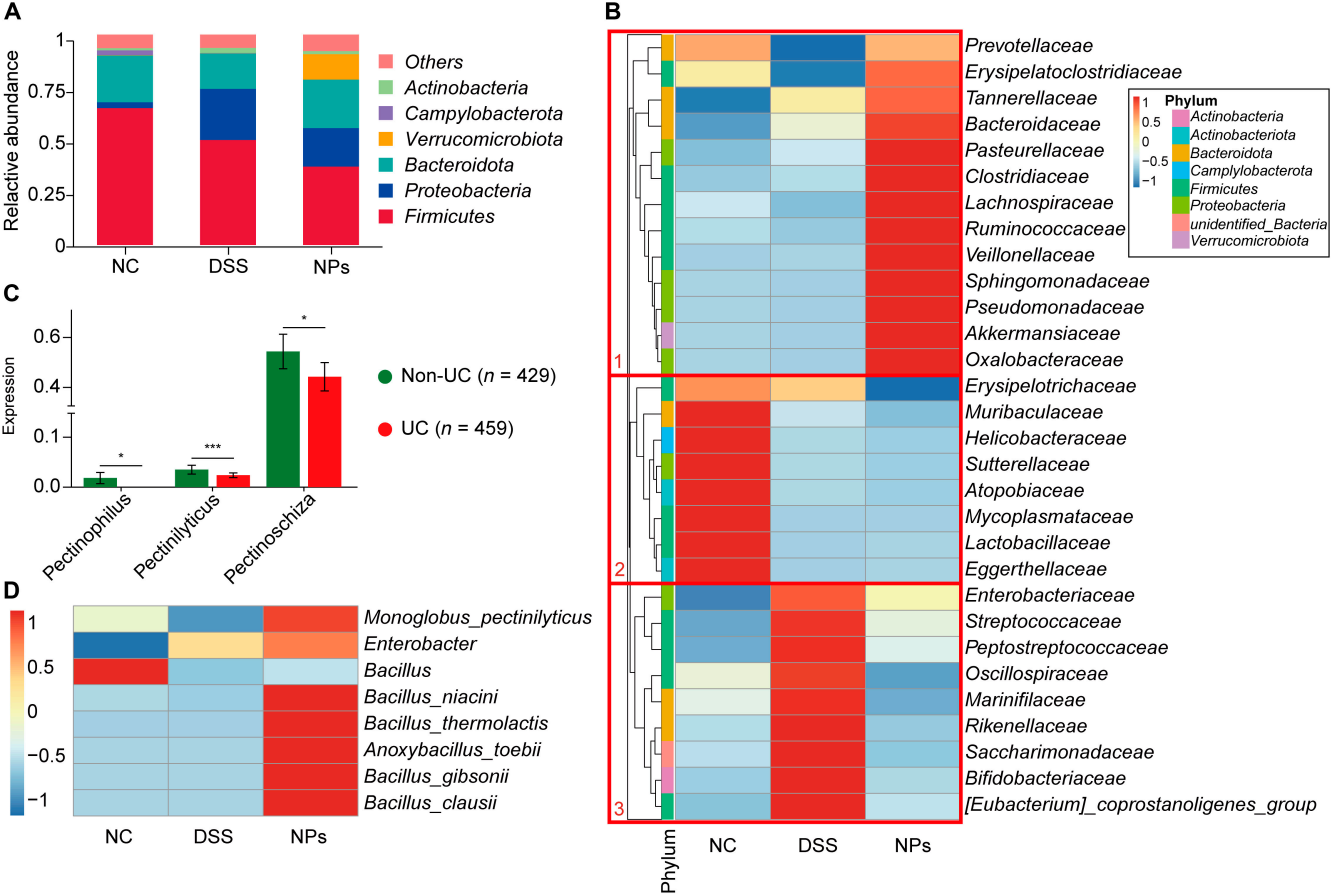
Alteration of gut microbiota by SAS@PLGA-Csn-Pcn NPs. The abundance of gut microbiota at the phylum level (A) and family level (B) (*n* = 4). (C) Comparison of relative abundance of pectinase-associated bacteria between healthy (*n* = 429) and UC populations (*n* = 459) based on metagenomic studies. (D) The relative abundance of pectinase-producing bacteria among 3 groups (*n* = 4). Compared with the UC population group, *P < 0.05, ***P < 0.001.

The treatment of NPs also significantly adjusted the composition of gut microbiota but did not simply reverse the changes caused by DSS (Fig. 7). DSS treatment significantly changed the composition of gut microbiota at the phylum level (Fig. 7A). Compared with the NC group, DSS treatment significantly decreased the levels of *Firmicutes* and *Bacteroidota*. The supplementation of NPs significantly increased the level of *Verrucomicrobiota*. Then, the composition of the gut microbiota at the family and genus levels was analyzed for 3 groups (Fig. 7B and Fig. S1). Cluster 1 consists of beneficial bacteria including *Akkermansiaceae*, *Prevotellaceae*, *Bacteroidaceae*, and *Ruminococcaceae*, which were significantly increased by NP treatment. Cluster 3 was composed of harmful bacteria, such as *Enterobacteriaceae*, *Streptococcaceae*, *Peptostreptococcaceae*, *Oscillospiraceae*, and *Marinifilaceae*, which were significantly decreased by NP treatment (Fig. 7B). At the genus level, the bacteria in cluster 1 were reduced and the bacteria in cluster 2 were increased after DSS induction (Fig. S1). Furthermore, NP administration decreased bacteria in cluster 2, which are composed of harmful bacteria, including *Escherichia–Shigella*, *Streptococcus*, and *Romboutsia*. These findings further indicated that NPs can modify the relative abundance and overall composition of the gut microbiota. Pectinases represent a common group of Pcn degrading. As shown in Fig. 7C, compared to the healthy population group, we found that the relative abundance of bacteria associated with the production of pectinase increased in the UC population group, suggesting that Pcn metabolism may be deficient in UC patients. Meanwhile, the relative abundance of bacteria associated with pectinase in mice gut was analyzed and the heatmap was described (Fig. 7D). Compared with the DSS group, the abundance of *Bacillus_niacini*, *Bacillus_thermolactis*, *Bacillus_gibsonii*, and *Bacillus_clausii* that belong to the genus *Bacillus* was increasing in the NPs group, which was a highly efficient source of production of pectinase [24]. In summary, NPs have demonstrated the ability to alleviate DSS-induced colitis in mice by increasing the abundance of beneficial bacteria and decreasing the abundance of harmful bacteria.

## Discussion

Currently, the exact cause of UC remains incompletely understood. Nonetheless, the treatment strategies for UC patients mainly focus on limiting inflammatory mediators or inhibiting pro-inflammatory processes [25]. The healthy human gut is a home to over 100 trillion viable microbes, rendering it a distinct ‘‘organ’’ of the host [26]. The gut microbiota is implicated in the pathogenesis of UC, thereby exerting an influence on the inflammatory state of the patients [27]. Meanwhile, the dysbiosis of gut microbiota may result in the colonization and proliferation of pathogenic microorganisms (such as pathogenic bacteria), destroy the intestinal mucosal barrier, and thus aggravate UC symptoms [28]. In this study, the composition of PCDDSs comprises naturally occurring prebiotic shells, which can effectively modify the gut microbiota.

Due to the complexity of the GIT environment, effectively targeting drugs specifically to the inflamed site in the colon poses a major challenge for oral PCDDSs [6]. Recently, both extracellular vesicles (EVs) and synthetic NPs have a series of advantages as ideal drug candidate carriers [29]. In contrast to EVs, synthetic NPs have a precisely controlled size and shape that can change their surface properties to improve cellular uptake and drug release efficiency. In addition, synthetic NPs can also be engineered to carry multiple drugs, with a wider range of applicability [30]. In the present study, the strong electrostatic interaction between the Pcn/Csn shell enabled the formed NPs to exhibit efficient pH sensitivity, thereby enhancing protection against low gastric pH in the upper GIT (Fig. 2). Due to the controlled release behavior, NPs facilitated a prolonged process of adhesion, uptake, and transport of SAS through the GIT, thus delaying its absorption process (Fig. 2H and I). The results of cell uptake and adhesion evaluation showed increased adsorption capacity of NPs at the site of inflammation (Figs. 3 and 4). Consequently, our oral PCDDSs exhibited resistance to gastric degradation, made a smooth transition into the colon, selectively adhered to the inflamed locus, and subsequently underwent internalization by specific target cells within the distal colon, and owing to the controlled release behavior, they facilitated a controlled and sustained drug release.

Our PCDDSs are unique in that they enable precise control of drug release. The results indicated that the rate of release of NP-carried SAS is influenced by the extent to which pectinase secreted by gut microbes mediates Pcn decomposition in the colon (Fig. 2I). Meanwhile, the analysis of metagenomic data revealed a significant decrease in the pectinase production capacity of pectinase among bacteria from UC patients compared to healthy individuals (Fig. 7C). Simultaneously, our investigation revealed a notable augmentation in the abundance of typical pectinase-producing bacteria within the gut microbiota in the presence of prebiotic NPs (Fig. 7D).

In the context of intestinal mucosal immune abnormalities, the main pathogenic mechanism of UC is mainly related to Treg/Th17 cell-mediated adaptive immune response. Our work demonstrated that NPs maintain a favorable Treg/Th17 cell balance in DSS-induced colitis in mice (Fig. 4). Simultaneously, it was observed that NPs exhibited a more pronounced ameliorative impact on parameters including weight loss, colon length reduction, colon tissue integrity, and systemic inflammatory status, thus substantiating their therapeutic efficacy in colitis. In addition to their remarkable efficacy in UC treatment, NPs could self-assemble using only 2 natural components, chitosan and Pcn, thereby reducing any potential toxic side effects on the body (Fig. 3C).

The Pcn/Csn shell of NPs is widely acknowledged as a prebiotic, enhancing the gut microbiota profile and alleviating colitis symptoms in UC patients [31,32]. The results of gut microbiota analysis in this study showed that NP treatment could significantly improve the bacterial diversity and generally modify the gut microbiota composition (Figs. 6 and 7). Specifically, various beneficial bacteria were significantly increased by NP treatment including *Akkermansiaceae*, *Prevotellaceae*, *Bacteroidaceae* [33], and *Ruminococcaceae* [34] (Fig. 7B). It should be noted that *Prevotellaceae*, as a butyrate-producing bacterium, exhibits the capacity to suppress the M1 polarization of colonic macrophages and the production of associated pro-inflammatory factors such as IL-1β and TNF-α [35]. In addition, harmful bacteria, such as *Enterobacteriaceae*, *Streptococcaceae*, *Peptostreptococcaceae*, *Oscillospiraceae*, and *Marinifilaceae* [36–39] at the family level (Fig. 7B) and *Escherichia*–*Shigella*, *Streptococcus*, and *Romboutsia* [37,38,40] at the genus level (Fig. S1), were significantly decreased by NP treatment. Besides, as an additive in NPs, several studies have indicated that PLGA, a commonly used biodegradable polymer, can lower the local pH in the gut and influence gut microbiota [41]. These effects are primarily attributed to the degradation products of PLGA, such as lactic acid and glycolic acid [42], which can alter the gut environment and microbial composition. This acidic environment may favor the growth of acidophilic bacteria, such as lactobacilli, while inhibiting the growth of other microbes that prefer neutral pH [43]. In conclusion, those results indicated that PCDDS is a potential modulator of gut microflora. NPs appear to regulate the balance of Treg/Th17 in a gut microbiota-dependent way, including metabolites derived from functional bacteria or microbiota. Previous studies have demonstrated that *Allobaculum* facilitates the proliferation of Th17 cells in the GIT, whereas our findings indicate that NPs can attenuate the relative abundance of this specific microbiota [44]. *Clostridium butyricum* is a gram-positive anaerobic bacterium in the family *Clostridiaceae*. This bacterium has a dose-dependently protective effect on DSS-induced colitis, which is closely related to the down-regulation of IL-17 secretion and inhibition of toll-like receptor 2 (TLR-2) [45]. Furthermore, *C. butyricum* inhibits the expression of TLR-4, TLR-5, and nuclear factor kappa-B p65 (NF-κB p65), promotes the activation of inhibitor of kappa B alpha (IκBα), and synergizes with other negative regulators [46]. The genus *Akkermansia* has anti-inflammatory potential and interacts with TLR-4 to improve colitis through up-regulation of the RORγt^+^ Treg cell-mediated immune response [47]. Interestingly, the inhibitory effect of *Akkermansia muciniphila* on TLR also provided more in-depth research prompts [48,49]. Those findings demonstrate that NPs may modulate the Treg/Th17 balance through gut microbiota and are related to the expression of TLR signaling pathways, providing a direction for future in-depth research. Taken together, we have demonstrated the potential to treat UC by combining gut microbiota modulation with anti-inflammatory effects via PCDDSs. The Pcn/Csn shell provides pH-dependent protection in the upper GIT and serves as a colonic delivery system activated by gut microbiota enzymes, facilitating colon-specific targeted release. This precise and controllable PCDDS represent a promising approach for improving UC treatment.

## Data Availability

The original contributions presented in the study are publicly available. These data can be found at https://submit.ncbi.nlm.nih.gov/subs/sra/SUB13789277/overview, SUB13789277, accessed on 2023 December 31.
