# Supplementary material for: Combining Gut Microbiota Modulation and Enzymatic-Triggered Colonic Delivery by Prebiotic Nanoparticles Improves Mouse Colitis Therapy
Source: Biomater Res. 2024 Aug 13;28:0062. doi: 10.34133/bmr.0062 (PMC11321063; doi:10.34133/bmr.0062)
Supplement: Supplementary 1 — Fig. S1 Table S1 [file bmr.0062.f1.docx]

**Supplementary Materials**


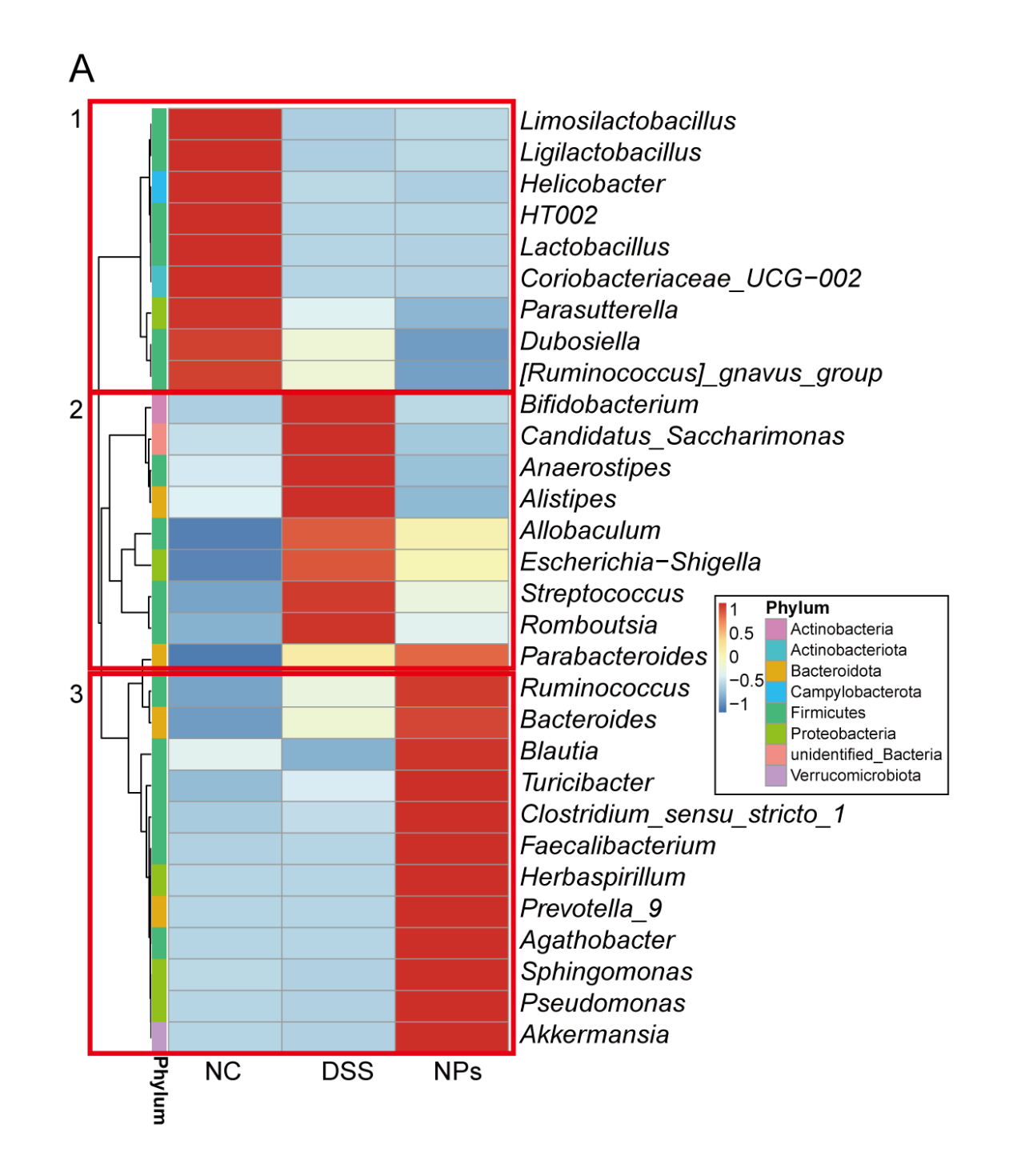


**Fig. S1.** Heatmap depicted the normalized abundance of each microbiota from fecal samples among NC, DSS and NPs group at the genus level.

**Table S1.** Assessment of the DAI index

| Weight loss (%) | Stool consistency | Occult/gross bleeding | Score |
| --- | --- | --- | --- |
| 0 | Normal | Normal | 0 |
| 1-5 | Loose stool | Hemoccult positive | 1 |
| 5-10 |  |  | 2 |
| 10-15 | Diarrhea | Gross bleeding | 3 |
| >15 |  |  | 4 |
